# Health communication strategies to address parental vaccine hesitancy regarding childhood vaccination in Nigeria: a systematic review

**DOI:** 10.3389/fpubh.2026.1854581

**Published:** 2026-08-25

**Authors:** Tarela Juliet Ike, Dung Ezekiel Jidong, Callistar Kidochukwu Obi, Maureen Iru Ntaji, Mulugeta Tamire, Marekegn Habtamu, Mieyebi Lawrence Ike, Evangelyn Ebi Ayobi, Peremi Richmond Ike, Rukevwe Francis Doghor, Rakhshi Memon, Kelvin Odeyovwi Ayobi, Aboneh Solomon Sime, Otovwe Agofure, Nusrat Husain

**Affiliations:** 1 Division of Psychology and Mental Health, University of Manchester, Manchester, United Kingdom; 2 School of Social Sciences, Humanities and Law, Teesside University, Middlesbrough, United Kingdom; 3 Department of Economics, Faculty of the Social Sciences, Delta State University, Abraka, Nigeria; 4 Department of Community Medicine, Delta State University Teaching Hospital, Oghara, Delta, Nigeria; 5 School of Public Health, Addis Ababa University, Addis Ababa, Ethiopia; 6 The Leprosy Mission International, Addis Ababa, Ethiopia; 7 Western Governors University, Salt Lake City, UT, United States; 8 Manchester Global Foundation, London, United Kingdom; 9 Tare Wyd Legal Chambers, Warri, Nigeria; 10 Beryl Foundation, London, United Kingdom; 11 University College London, London, United Kingdom; 12 Department of Public Health, University of Delta, Agbor, Delta, Nigeria; 13 Mersey Care National Health Services (NHS) Foundation Trust, Liverpool, United Kingdom

**Keywords:** childhood immunization, health communication, Nigeria, parents, strategies, vaccine hesitancy

## Abstract

**Background:**

Childhood vaccine hesitancy is a significant public health concern. In Nigeria, it remains a major barrier to achieving optimal immunization uptake, contributing to preventable morbidity and mortality. Yet there remains a significant research gap on the synthesis of health communication strategies to address parental hesitancy to childhood vaccination. The primary aim and original contribution of the present review is to synthesize health communication strategies to address parental hesitancy to childhood vaccination in Nigeria.

**Method:**

The review strategy was underpinned by the Population, phenomenon of interest and context (PICo) model. Hence, the population (parents, carers, or primary caregivers of children between the ages of 0 and 3 years), the phenomenon of interest (health communication strategies to address parental hesitancy to childhood vaccination), and the context (vaccine hesitancy). For qualitative studies, the PICo strategy, which is a modified version of PICO, was used. Boolean operators (AND/OR/NOT) were used to ensure rigor in adopting informed search terms. Six databases were searched for published articles between 2017 and March 2026. *N* = 12 studies met the inclusion criteria, and the relevant data were synthesized and analyzed using a thematic synthesis approach. The review protocol was pre-registered on the PROSPERO Registry (no. CRD420261303323).

**Findings:**

The synthesized studies and analyses yielded the following main findings: (i) Media campaign strategies, (ii) State-sponsored communication strategies, (iii) Community-centered strategies, and (iv) Phone-based strategies.

**Conclusion:**

Childhood vaccine hesitancy remains a major challenge in Nigeria. The review recommends that vaccine communication be culturally attuned and multi-channeled (e.g., embedding community-centered mechanisms—particularly those involving trusted religious and traditional leaders, media platforms, and mobile phone text messaging). It also recommends that vaccine communication be responsive to both structural constraints and community information need to address parental hesitancy to childhood vaccine uptake.

## Introduction

Parental hesitancy to childhood vaccination, defined as a refusal or delay in accepting vaccines despite their availability (1), affects an estimated 1 in 5 (about 21%) parents of children aged 0–6 years globally (2). Globally, infectious diseases are a significant cause of under-five mortality. In low- and middle-income countries (LMICs), vaccine-preventable diseases account for a considerable proportion of these deaths (3). More specifically, worldwide, sub-Saharan Africa has the highest under-five mortality rates, and in Nigeria, vaccine-preventable diseases contribute significantly to these (4). Nigeria is also one of the countries accounting for the highest number of children under-immunized globally, with an estimated 18%−29% of infants missing vital vaccines and approximately 2.1 million children unvaccinated in 2024 (5). According to the World Health Organization (6), this is despite having a significant number of children under 1 year old—the country still struggles with poor rates of immunization in the sub-Saharan region. Yet, a gap remains in the synthesis of health communication strategies to address parents' and carers' childhood vaccine hesitancy and encourage uptake.

Global evidence suggests a growing number of parents and caregivers delaying or refusing childhood vaccinations (2, 7). This growing hesitancy is largely fueled by declining confidence in the benefits of vaccines, distrust in health sectors delivering vaccines (8), limited understanding of vaccine-preventable diseases (9), and the misconception that immunization is less necessary than public health guidance suggests (10). Such delays and refusals obstruct communities and entire countries from achieving the vaccination level required for herd immunity, leaving populations vulnerable to outbreaks whenever a vaccine-preventable pathogen begins to circulate (11). For any vaccination program to function effectively, a consistently high level of vaccine uptake is crucial (12), as it is the only way to maintain strong herd protection.

Within the context of Nigeria, childhood vaccine hesitancy is driven by a complex interplay of informational, sociocultural, and systemic factors (59). Numerous studies highlight that low trust in vaccine safety and the healthcare system, longstanding mistrust of Western medical interventions and widespread misinformation continue to undermine parental confidence in routine immunization (13). For example, qualitative research among Nigerian mothers found low trust in vaccine safety and health providers, alongside strong religious beliefs that place ultimate responsibility for child health on divine protection, leading them to view vaccines as less essential (14). Historical skepticism in regions such as Kano, where parental hesitancy is shaped by concerns about foreign influence and misperceptions about vaccine intentions, further intensifies reluctance (15). Additionally, cultural and linguistic barriers, entrenched myths, and persistent misinformation—particularly in northern states—heighten uncertainty and fuel behavioral resistance to vaccination (16, 59).

Furthermore, given Nigeria's patriarchal nature (17, 18), structural and gender-related barriers also significantly contribute to poor childhood vaccination uptake. Evidence from both UNICEF and academic research identifies gender-skewed decision-making, where fathers often hold primary authority over healthcare decisions, limiting mothers' ability to vaccinate their children even when willing (19). Socioeconomic constraints—including poverty, competing household priorities, and vaccine-related costs or access challenges—further reduce vaccination completion, particularly in underserved communities. Past negative experiences with health services, limited vaccine availability in some regions, and geographic disparities create an environment in which parents either delay or avoid immunization (14). Moreover, a recent systematic review shows that religion, ethnicity, household wealth, maternal education, and rural residence strongly influence childhood immunization inequities across Nigeria, reinforcing patterns of hesitancy and low uptake (20).

Despite the preceding literature, a gap remains. This systematic review therefore makes an original and significant contribution by synthesizing existing evidence on non-clinical community-based strategies to address parents and carers' childhood vaccine hesitancy and encourage uptake in Nigeria. The review was also informed by the Health Belief Model (HBM) (21, 22), which provides a valuable analytical lens for reviewing non-clinical strategies aimed at reducing childhood vaccine hesitancy in Nigeria. By focusing on how strategies shift key beliefs—perceived disease risk, perceived vaccination benefits, perceived barriers, and cues to action—the HBM helps identify which strategies effectively counter misinformation, build trust, and motivate parents and carers to vaccinate. Using this framework allows the review to pinpoint what drives behavior change, clarify why certain approaches improve uptake and reduce preventable child deaths, and highlight where more targeted, community-responsive strategies are needed. Hence, the research question:

What are health communication strategies to address parental hesitancy to childhood vaccination in Nigeria?

The review commences with a report on the methodology. This is followed by the findings and discussion. The review concludes and makes recommendations for policy designed to improve vaccine uptake in Nigeria.

## Method

### Protocol/search strategy

The review protocol was guided by the Preferred Reporting Items for Systematic Reviews and Meta-Analyses (PRISMA), a framework that strengthens methodological rigor, enhances reproducibility, and ensures transparent reporting throughout the review process (23). Before commencing the review, a detailed protocol was developed outlining the inclusion and exclusion criteria as well as the analytical procedures to be used, in accordance with established guidance for systematic review design (24). To further reinforce transparency and accountability, the protocol was prospectively registered with the PROSPERO international database (Registration No: CRD420261303323).

### Study's inclusion and exclusion criteria

The review's main aim was to identify and synthesize health communication strategies to address vaccine hesitancy among Nigerian parents. Therefore, the search terms were designed to ensure an in-depth body of literature relevant to the review. Hence, the inclusion criteria: (i) Mixed-methods, qualitative, or quantitative studies of any design examining health communication strategies aimed at reducing childhood immunization vaccine hesitancy in Nigeria. (ii) Studies adopting cross-sectional studies, observational studies, longitudinal studies, cohort studies, and population-based surveys. (iii) Full-text publications only, including peer-reviewed articles and other academic sources. (iv) Studies involving primary caregivers from populations affected by childhood immunization vaccine hesitancy. (v) Studies conducted exclusively within Nigeria. (vi) Studies assessing any form of strategies targeting vaccine hesitancy, including Community-based strategies, Media or communication campaigns, educational strategies, psychosocial strategies (e.g., psychoeducation, community advocacy) and (vii) Studies including parent or primary caregivers affected by vaccine hesitancy. The study also focused on communication strategies targeting recommended vaccines for children aged 0–3. This include Bacillus Calmette-Guérin (BCG) vaccine, Oral Polio Vaccine (OPV), Rotavirus vaccine, Meningitis vaccine, measles vaccine, and Pentavalent vaccine. It also includes Pneumococcal Conjugate Vaccine (PCV), Malaria vaccine, yellow fever vaccine, Diphtheria Tetanus and Acellular Pertussis (DTaP) vaccine, Hepatitis A, and Typhoid vaccines (25–27).

The review excluded the following: (i) studies that do not examine strategies aimed at addressing childhood immunization vaccine hesitancy in Nigeria. (ii) Studies where the population does not focus on primary caregivers and/or children. (iii) Unpublished works such as poster abstracts, blogs, or conference abstracts. (iv) Studies not published in English and without an available English translation. (vii) Studies that do not meet the predefined inclusion criteria for this review.

### Search strategy

The review was underpinned by the PICo framework, which defined the Population as Parents whose children are not up to date with vaccination, the Phenomenon of Interest as health communication strategies, and the Context as addressing parental childhood vaccination hesitancy (28). In addition, Boolean operators (AND, OR, NOT) were used to construct precise and strategic search terms to enhance the effectiveness of the literature search (29). Below are examples of search terms adopted, as shown in Table 1.

**Table 1:** Sample search terms.

Data base	Search terms	Date searched	Result returned
PubMed	• (“Mothers”[MeSH] OR “parents”[tiab] OR “fathers”[MeSH] OR “caregivers”[tiab] OR “Childhood vaccine hesitancy”[MeSH] OR “vaccine hesistancy”[tiab] OR “vaccine resistance”[MeSH] OR “vaccine scepticism”[tiab] OR “Nigeria”[tiab] OR “North”[tiab])	28/02/2026	64,976
	• (“Parents”[MeSH] AND “Carer” [tiab] OR “Caregiver” [MeSH] OR “childhood vaccine hesitancy”[tiab] OR “childhood vaccine delay”[MeSH] OR “Nigeria”[tiab])		4,459
	• (“Intervention”[MeSH] AND “vaccine hesitancy”[tiab] AND “Nigeria”[MeSH], AND “Kano” [tiab] AND “Katsina” [tiab] AND “Plateau” [tiab] OR “parents” AND “child” [tiab] AND “childhood immunization” [tiab] AND “Nigeria” [tiab])		3
CINAHL	• “Vaccine hesitancy intervention” AND “Nigeria” NOT “Canada” OR “United Kingdom” OR “Asia” OR “USA” AND “nonclinical intervention addressing vaccine hesitancy” OR “educational intervention” “Media campaign intervention”	4/03/2026	84, 548
Cochrane Controlled Trial Register	• “Media campaign intervention” OR “community-based intervention” OR “art-based intervention” AND “parent” AND “Vaccine hesitancy”	4/03/2026	9,311 (trials) 100 (Cochrane reviews)
	• “primary care givers” OR “childhood vaccine uptake intervention” OR “public health intervention” AND “Nigeria” OR “Adamawa”		528 (trials) 11 (Cochrane reviews)

The search strategy informed the systematic exploration of six major databases: PubMed, CINAHL, Applied Social Science Index and Abstracts, Embase, PsycInfo, and Cochrane Controlled Trial Register. These database searches were supplemented by manual screening of the reference lists of included studies and by additional searches conducted through local academic libraries, including the Teesside University Library in Middlesbrough. To ensure the relevance and currency of the evidence base, the review synthesized studies published between 2017 and March 2026.

### Included studies screening and selection

A total of 183,936 records were identified across the six databases, and 14,700 were identified through other comprehensive searches, such as Google Scholar and relevant studies' reference lists. Cumulatively, the total from both the database and other comprehensive searches = 198,636. Of the 198,636 studies initially retrieved, 198,425 duplicates and non-relevant studies were removed. The remaining records then underwent a rigorous two-phase screening process. First, two independent reviewers assessed the titles and abstracts against the predefined inclusion and exclusion criteria. Studies that lacked empirical data, did not focus on the target population, or were clearly irrelevant to the scope of the review were excluded at this stage.

**Figure 1:**
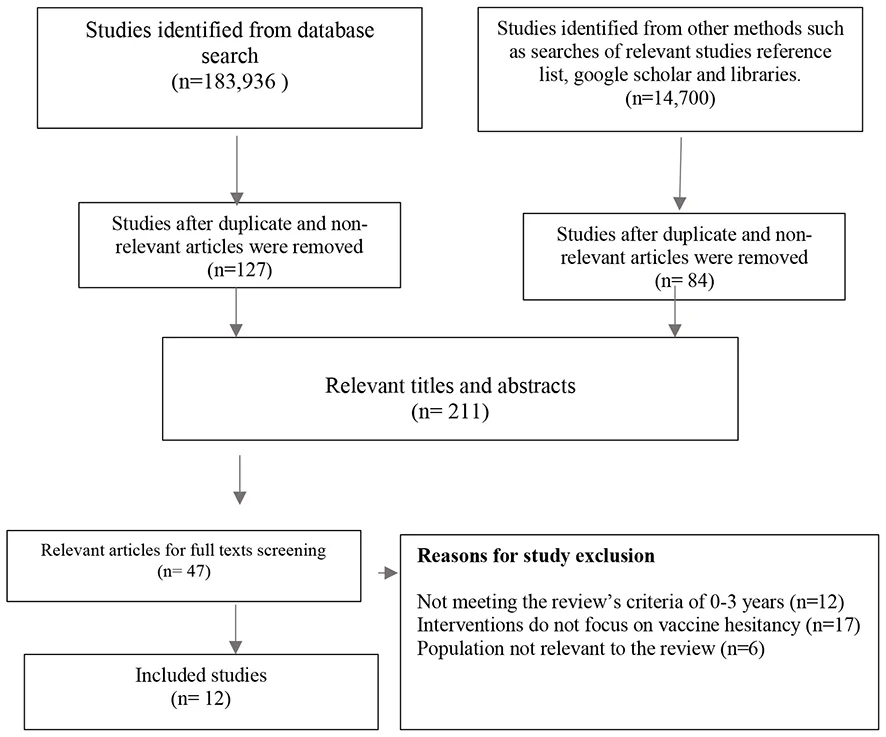
PRISMA flow diagram showing trajectory of the literature review process.

Following this initial screening, 47 studies were deemed eligible for full-text review. The same two reviewers independently evaluated each full-text article, applying the review's inclusion criteria consistently and with methodological rigor. Any discrepancies in decisions were resolved through consultation, discussion, and consensus among the review team to ensure objectivity and reliability. In total, 12 studies met the inclusion criteria and were incorporated into the final synthesis. A detailed PRISMA-style flow diagram summarizing the screening process, including numbers of excluded and included studies at each stage, is presented in Figure 1. Reviewers further evaluated the relevant studies to ensure they met the criteria. Where discrepancies existed, they were addressed through consultation and discussion among the review team.

### Assessment of risk of bias

Qualitative studies that met the review's inclusion criteria were appraised using the Joanna Briggs Institute (30) Critical Appraisal Checklist for Qualitative Research, a 12-item tool designed to evaluate methodological rigor. The checklist includes questions such as: IS there congruity between the research methodology and the interpretation of results? And is there congruity between the stated philosophical perspective and the research methodology? Each item is rated using the response options Yes, No, Unclear, or Not applicable.

Mixed-methods studies were evaluated using the Critical Appraisal Skills Program (31) checklist. CASP provides a structured set of 10 quality-assessment questions, including: WAS the data collected in a way that addressed the research issue? And was the chosen qualitative/quantitative method appropriate? The tool allows reviewers to record responses—yes, no, or can't tell—and document the rationale for each decision. CASP also supports the assessment of potential bias within the qualitative components of mixed-methods designs. Overall, studies assessed with CASP demonstrated a low risk of bias (see Appendix Table A4).

Randomized controlled trials were evaluated using the JBI Critical Appraisal Checklist for Randomized Controlled Trials, which comprises 14 items and assesses core elements of trial validity. For quantitative studies more broadly, the Symonds and Tang (32) Quality Appraisal Checklist was used to determine risk of bias. See the Appendix for the full risk-of-bias assessments for all studies included in the present review.

## Data synthesis

The key findings from the included studies were thematically synthesized to address the central research question. The synthesis process was guided by Toracco's (33) conceptual framework for integrative reviews, which emphasizes the importance of grounding evidence synthesis in a coherent theoretical foundation. In this review, Kimberlé's (58) intersectionality theory served as the guiding conceptual lens, shaping both the organization of the evidence and the critical interrogation of each study's methodological and conceptual contributions.

A synthesis matrix was used to support the systematic evaluation of all included studies, enabling the clear identification of methodological strengths, weaknesses, and gaps across the evidence base. The synthesis proceeded through a four-stage analytical process: (i) conducting a comprehensive and methodologically robust literature search; (ii) extracting and identifying key concepts and thematic elements; (iii) organizing these concepts to map relationships, divergences, and points of convergence across studies; and (iv) integrating and synthesizing the evidence to determine implications for future research and articulate the need for further health communication strategy-oriented inquiry.

The synthesis matrix facilitated a structured organization of sources, mapping studies against emerging themes to visually illuminate patterns, debates, and areas of conceptual sparsity. To enhance analytical rigor, an index-card method was employed during the fourth stage. Key findings from each study were summarized on individual cards, categorized by theme, and arranged to form a visual conceptual map. This process supported the identification of cross-cutting linkages and evidential gaps, which directly informed the development, sequencing, and articulation of the review's findings.

## Results

Based on the thematic synthesis of relevant studies, the following themes emerged: (i) Media campaign strategies, (ii) State-sponsored communication strategies, (iii) Community-centered strategies, and (iv) Phone-based strategies. Below is a table summarizing each theme and corresponding studies (Table 2).

**Table 2:** Media campaign strategies.

S/n	Themes	Studies
i	Media campaign strategies	(34–39)
ii	State sponsored communication strategies	(34–36, 40)
iii	Community-centered strategies	(34–39, 41, 42)
iv	Phone based strategies	(40, 43–45)

### Media campaign strategies

Several studies reported the use of media campaign strategies, such as indigenous communication media (34), radio (35, 36), mass media (37, 38) and audio messaging strategies designed to inform culturally relevant messages on infant and maternal knowledge relating to vaccination knowledge, attitudes and willingness to engage in vaccine uptake among rural women (39). For example, Iwu et al.'s (39) study, comprising 193 respondents who took part in the testing of a culturally tailored vaccination message for children under the age of 5 and pregnant women, found that while knowledge scores increased significantly post-intervention (*Z* = 4.155, *p* < 0.001, *r* ≈ 0.30), indicating a moderate effect, willingness to vaccinate remained unchanged (*p* = 0.809). Another study, a cross-sectional survey exploring the use of indigenous communication media to promote immunization among mothers in rural communities in Abia State, Nigeria, found a statistically significant relationship between the use of indigenous media and vaccination, including immunization communication (34). The study also found that (73.6%) of the respondents were of the view that media incorporating indigenous communication are highly relevant in informing rural dwellers about immunization programs (34).

### State sponsored communication strategies

A number of studies reported the use of state-sponsored communications strategies using a range of mechanisms, including primary health care (35, 37), health education sessions conducted at other health facilities involving immunization or antenatal clinics (36) or the use of health visits (40). While communication strategies could play a pivotal role in educating participants about vaccines, the type of media campaign could also determine its potential efficacy. One study drawing on a mixed-methods design, which assessed the Ministry of Health's health communication strategies in Childhood Immunization Campaigns in some States of North Central Nigeria, found that awareness of the child immunization campaign was due to the role played by influential traditional and religious leaders, and that illiteracy and ignorance were major challenges of the child immunization campaign (37). It also found no significant difference in the health communication strategies adopted by the Ministry of Health in the child immunization campaign in the North-Central states of Plateau, Nasarawa, and Benue, and that these strategies were perceived to be largely ineffective (37).

### Community-centered strategies

Several studies reported using a community-centered approach. This includes traditional and religious leaders (38, 41, 42), town criers [e.g., indigenous communication media (34), and local music strategies promoting vaccine messages (39)]. Others adopted strategies, such as religious gatherings, to influence caregivers' acceptance of vaccine uptake (35, 37) and parents' and caregivers' preference for vaccines (36, 38). One study used a descriptive survey to evaluate the communication strategies used by the Niger State Primary Health Care Development Agency in Nigeria to mitigate polio vaccine hesitancy. Based on data from 95 caregivers regarding campaign influence, channels, exposure, and attributes, the study found that religious gatherings and radio broadcasts were highly effective in reducing hesitancy (35). However, message timing inconsistencies and limited integration of digital channels hinder sustained impact, particularly in improving vaccine uptake (35). Another study tested the training of health workers and religious and traditional leaders on vaccination, their leadership tasks, and community mobilization in Calabar (41). The study evaluated the strategy among 2598, 2570, and 2550 children who were assessed at baseline, midterm, and endline, respectively. The study found no evidence that the strategy affected the proportion of children up to date with vaccination (*p* = 0.69) (41). However, the strategy showed effectiveness in reducing unvaccinated numbers of children from 7 to 0.4% (*p* = 0.001) alongside the timeliness of the later vaccines Penta 3 (OR 1.55; 95% CI: 1.14, 2.12; *p* = 0.005) and Measles (OR 2.81; 96% CI: 1.93–4.1; *p* < 0.001) (41).

Another study explored 84 caregivers' perspectives in Bauchi and Cross River states on avenues for effective vaccination communication with parents to overcome barriers to childhood vaccine hesitancy and found that town announcers, churches/mosques, and media announcements were the preferred choices (36). The study also found that the clinic environment, health worker attitudes, and long waiting times are significant barriers to receiving vaccination information and uptake (36). A similar finding was reported in a study that surveyed 97 men and 101 women in the northern region regarding their preferences for strategies to improve childhood vaccine uptake (38). The study found that involving religious leaders and disseminating information through media campaigns were among the preferred strategies (38).

### Phone based strategies

A number of studies reported the use of phone calls (43) and mobile phone text message strategies to encourage vaccine uptake and address childhood vaccine hesitancy (40, 44, 45). Yunusa et al. (44) conducted a quasi-experimental study to assess the effects of text messages sent via mobile phones and call reminders on the completeness of vaccination against pertussis, diphtheria, hepatitis B, tetanus, and *Haemophilus influenzae* in Kano State, Nigeria. Based on data from 541 mothers (271 in the strategy group and 270 in the control group), the study found that completion rates for the three doses of the vaccine were observed to be higher for children in the strategy group (*n* = 161, 59.4%) compared to the control group (*n* = 92, 34.1%) (44). The study concludes that mobile phone reminders were effective in increasing the completeness of childhood vaccination (44). Another study tested the effectiveness of Mobile Phone reminders and Home Visit outreach in improving vaccination among children under 2 years of age. Three hundred and eighty-four participants were randomly assigned to either the mobile phone, home visits, or no strategies control group, and the study found that both strategies were effective in improving vaccination uptake, with a statistically significant difference (*p* = 0.015) and a moderate effect size (0.257) (40). However, home visit outreach recorded the highest completion rate (40).

## Discussions

A total of 12 studies met the review's inclusion criteria. To ensure methodological rigor, two independent reviewers conducted a comprehensive risk-of-bias assessment, evaluating both the credibility of each study and the strength of the overall evidence base. This process involved identifying potential sources of error in study design, implementation, or analysis that could compromise the reliability of the results. Applying a structured risk-of-bias framework to each study (see Appendix) strengthened transparency and enabled clearer judgment of confidence in the findings, supporting more informed decision-making in practice and in guideline development. The synthesized studies yielded several themes, including media campaign strategies, state-sponsored communication strategies, community-centered strategies, and phone-based strategies.

A recurrent theme our review found was the prevalence of the use of media campaign strategies. This includes indigenous communication media and audio messaging strategies, such as radio, in 6 out of the 12 studies (34–39). The finding highlights the significance of media in encouraging vaccine uptake and reducing hesitancy. However, a significant finding is that media messages that adopt a culturally appropriate indigenous communication style are often perceived as more effective at informing parents and carers about vaccines and reducing hesitancy.

Another significant finding is the use of state-sponsored communication strategies involving primary health care (35, 37). State-sponsored communication strategies—delivered through primary healthcare structures and mass media—emerged across the reviewed studies as important but often inconsistently effective tools for promoting childhood immunization (35, 37). Although government campaigns are designed to educate caregivers about vaccines, evidence indicates that their impact is heavily shaped by the communication channels used and the extent to which messages align with community realities. As shown in Mark, Rabiu and Santas' study, awareness of immunization campaigns was largely driven not by formal state messaging but by trusted religious and traditional leaders, while widespread illiteracy and low health literacy limited the effectiveness of official communication efforts. The finding that state-led strategies were perceived as largely ineffective across multiple North-Central states mirrors wider research showing that top-down, generic communication campaigns often fail to shift vaccine behaviors unless they are locally tailored, culturally resonant, and delivered through trusted intermediaries. Consistent with global evidence (46, 47), these results underscore that successful vaccine communication requires integrating state messaging with community-embedded actors and adapting strategies to context-specific barriers, such as literacy levels and misinformation. However, in low- and middle-income countries such as Pakistan, a recent study found that major TV networks and doctors were trusted to address parental hesitancy and promote polio vaccine uptake among parents of children under 5 years old (48).

Furthermore, our review found that eight out of 12 studies adopted a community-centered approach, such as traditional and religious leaders, including religious gatherings to influence caregivers‘ acceptance of vaccine uptake and its preferences by parents and caregivers (33, 35–38, 41, 42). The evidence from the reviewed studies demonstrates that community-centered strategies—particularly those that leverage traditional and religious leaders, faith-based gatherings, and local communication networks—play an influential role in shaping caregivers' attitudes toward childhood vaccination. Across multiple settings, religious gatherings, town announcers, and mosque/church communications consistently emerged as trusted and effective channels for addressing vaccine hesitancy (35, 36, 38). The effectiveness of these culturally embedded communication methods was further supported by evidence showing that radio broadcasts and in-person community engagement can significantly reduce hesitancy, although inconsistent message timing and weak integration of digital tools may limit long-term impact (35). Notably, while capacity-building for health workers and community leaders in Calabar did not improve the proportion of fully vaccinated children (41), the strategy did substantially reduce the number of completely unvaccinated children and improve timeliness for critical vaccines, suggesting that community-centered strategies may be particularly effective for reaching previously disengaged populations. Findings across studies also highlight persistent structural barriers—such as unfavorable clinic environments, poor health-worker interactions, and long waiting times—that undermine vaccination communication and uptake despite strong community support mechanisms (36). Taken together, these studies underscore that community-rooted approaches are highly valued and can yield meaningful improvements in vaccine acceptance, but their success depends on consistent messaging, integration with broader communication strategies, and parallel improvements in health-system responsiveness.

Finally, we found that four of 12 studies reported using phone-based strategies (40, 43–45). Evidence from the included studies demonstrates that mobile phone–based reminders are a promising strategy for improving childhood vaccination uptake in Nigeria. Both quasi-experimental and randomized designs show that SMS and call reminders significantly increase completion rates for multi-dose vaccines, with reminder groups achieving markedly higher adherence than controls (40, 44). These findings align with wider global evidence showing that mobile health (mHealth) strategies—particularly SMS reminders—can reduce missed appointments, strengthen caregiver recall, and improve routine immunization in countries, including the United States (49), Europe (50), and low- and middle-income settings (50, 52). A recent study by Delavande et al. (51) in Pakistan found that phone-based maternal support increased childhood immunization by 7% within a one-year period. Even within the context of the African continent, Gilano et al.'s (52) systematic review and meta-synthesis of mHealth strategies for childhood vaccination in Africa, comprising four quasi-experiments and 14 randomized controlled trials, found that their applications could improve childhood vaccination on the continent. It also found that, given the heterogeneity of the included studies, mobile health strategies are more effective in less-developed regions and when an additional incentive is included with the messaging system (52). Such incentives are also illustrated through the superiority of home-visit outreach in Promise et al.'s (40) study, which echoes broader literature suggesting that while digital reminders are effective, they may be most impactful when combined with personalized, community-based follow-up. Overall, the literature supports a blended approach where digital tools complement, rather than replace, interpersonal outreach to maximize sustained vaccine uptake.

## Implications

The findings from these studies underscore the need for vaccine communication strategies that are both context-responsive and multilayered. The consistent success of community-centered approaches—particularly those involving trusted religious and traditional leaders—suggests that future strategies must prioritize locally embedded structures rather than rely solely on state-led or generic mass-media campaigns. At the same time, the demonstrated effectiveness of mobile phone reminders highlights the value of integrating low-cost digital tools to enhance caregiver recall and support timely vaccine completion. However, the limited impact and perceived ineffectiveness of state-sponsored communication strategies (34–36, 40) appear to suggest that top-down messaging alone is insufficient without careful tailoring to local literacy levels, cultural norms, and caregivers' communication preferences. These findings align with wider global literature, which emphasizes that successful vaccination programs depend on combining community trust, personalized engagement, and consistent, well-targeted messaging (38, 53). For policymakers and practitioners, this means designing hybrid communication models that strengthen both community-led dialogue and digital outreach, while simultaneously addressing structural barriers such as health-worker attitudes, clinic environments, and message timing. Such an integrated approach is likely to yield more sustainable improvements in vaccine uptake and reduce persistent pockets of childhood vaccine hesitancy.

## Strengths and limitations

Our review acknowledges some limitations. Despite offering important insights into communication strategies for improving childhood vaccine uptake, the evidence reviewed is constrained by several methodological and contextual limitations. Many of the studies relied on relatively small or region-specific samples, limiting generalizability beyond the specific states or communities examined. The predominance of quasi-experimental and cross-sectional designs also restricts causal inference, and in some cases, the absence of long-term follow-up prevents conclusions about the sustainability of strategies such as SMS reminders or community outreach. Several studies relied on self-reported data from caregivers, which are vulnerable to recall and social-desirability biases—particularly in contexts where vaccination is socially expected. In addition, variations in strategy delivery, message quality, and implementation fidelity across studies make direct comparisons challenging and may partly explain the inconsistent effectiveness of state-sponsored campaigns. Importantly, structural factors such as health-system quality, digital access, and socio-economic inequalities were seldom measured systematically, even though these contextual variables likely shaped strategy outcomes. These limitations suggest that, while the findings provide valuable directional evidence, stronger, more rigorously designed evaluations are needed to fully understand which communication strategies are most effective, for whom, and under what conditions.

## Policy recommendations

To effectively reduce childhood vaccine hesitancy and strengthen routine immunization outcomes, first, policymakers are encouraged to adopt a multi-layered communication strategy. Multilayered refers to diverse communication strategies that incorporate both government- and community-led approaches (54). This strategy is also encouraged to include the institutionalization of community-led engagement structures and the formal integration of religious leaders, traditional authorities, and community influencers into immunization communication plans. As the review shows, these actors appear to demonstrate higher credibility and stronger reach than formal state messaging (34–39, 41, 42). Their involvement should be supported through structured training, incentives, and ongoing collaboration. Second, government and health agencies should scale up low-cost digital strategies such as SMS reminders and voice calls across primary healthcare systems. The effectiveness of mobile reminders in improving vaccine completion rates highlights the potential of mobile technology to complement interpersonal outreach, particularly in urban and semi-urban areas.

Thirdly, state-sponsored communication campaigns are encouraged to be context-specific and to more closely engage local stakeholders, such as community actors and local leaders, to dispel the low awareness associated with parental hesitancy toward childhood vaccination (37). Campaigns are also suggested to prioritize local languages, culturally appropriate formats, and communication channels that caregivers actively use, such as radio, town announcers, and faith-based gatherings. Fourth, policymakers are encouraged to address structural barriers within health facilities, including long waiting times, unfavorable clinic environments, and negative health-worker interactions, all of which undermine the impact of communication strategies. Finally, an integrated, multi-channel communication model is encouraged—one that combines community trust, digital reach, and responsive state leadership. This model should be embedded within national and state immunization strategies, supported by dedicated funding, testing its effectiveness and cost effectiveness via randomized controlled trial, alongside monitoring systems, and partnerships with civil society and media organizations. Such an approach would not only enhance vaccine uptake but also strengthen long-term public confidence in immunization programs.

## Conclusion

Parental hesitancy to childhood vaccination remains a global public health concern and requires multifaceted, culturally appropriate strategies to encourage uptake. Overall, the findings demonstrate that no single communication strategy is sufficient to address the complexity of childhood vaccine hesitancy; rather, a combination of locally grounded, technologically supported, and institutionally coordinated approaches is required. Community-centered mechanisms—particularly those involving trusted religious and traditional leaders—proved consistently influential in shaping caregivers' decisions, highlighting the centrality of social trust in vaccine acceptance. Mobile phone reminders emerged as a practical, scalable tool for improving vaccine timeliness and completion, though their effectiveness is enhanced when complemented by interpersonal outreach. In contrast, state-sponsored communication efforts were often viewed as generic and less impactful, especially in settings where literacy barriers and weak health-system engagement limited the reach and credibility of messages. Taken together, the evidence underscores that successful vaccine communication must be culturally attuned, multi-channel, and responsive to both structural constraints and community information needs. These insights provide a strong foundation for policy recommendations to strengthen Nigeria's immunization communication ecosystem and reduce persistent pockets of vaccine hesitancy.
