# Supplementary material for: Health communication strategies to address parental vaccine hesitancy regarding childhood vaccination in Nigeria: a systematic review
Source: Front Public Health. 2026 Aug 25;14:1854581. doi: 10.3389/fpubh.2026.1854581 (PMC13552396; doi:10.3389/fpubh.2026.1854581)
Supplement: Supplementary file 1 [file Supplementary_file_1.docx]

**Appendix 1: Study Characteristics**

| Author(s) | Aims | Population | Methods | Findings | Strengths | Limitation(s) |
| --- | --- | --- | --- | --- | --- | --- |
| Brown & Oluwatosin, (2017) | The study tested the acceptability and adaptability of immunization reminder/recall system in southwest Nigeria. | 595 mothers/infants pairs | Cluster randomized controlled trial. | The study found that immunization compliance rate was 79.2% among the children in the intervention group compared to 46.4% in the control group (p < 0.001). | The study tested the feasibility of the intervention. | The study was conducted in an urban area, and results may not be generalizable to peri-urban and rural settings. |
| Iwu et al., (2025) | This study explored the use of culturally adapted local music, to deliver relatable immunization messages. The aim was to assess the impact of culturally tailored audio messages on maternal and infant vaccination knowledge, and willingness to engage in vaccine uptake among rural women. | 20 caregivers in the qualitative arm and 193 respondents in the quantitative arm from Imo state. | Mixed method design. | Qualitative findings (N=20) revealed low perceived susceptibility to vaccine-preventable diseases, misconceptions about vulnerability, reliance on traditional remedies, and barriers such as mistrust, limited access, and gender dynamics. In the quantitative phase (N=193), knowledge scores increased significantly post-intervention (Z=4.155, p=0.809). | Involved developed and testing of culturally centred intervention. | Small sample size. |
| Kaka, Igvuve & Santas, (2026) | This study examined the communication strategies adopted by the Niger State Primary Health Care Development Agency  (NSPHCDA) to address polio vaccine hesitancy among caregivers in Minna state. | 95 caregivers. | Descriptive survey design. | The study found that radio  broadcasts and religious gatherings were highly effective in reducing hesitancy, fulfilling the first research objective. However, inconsistencies in message timing and limited integration of digital  channels hinder sustained impact, particularly regarding improving vaccine uptake. | Draws on data from caregivers. | The study data was based on perception-based surveys and responses  may reflect social desirability or recall bias. The study also focused on a specific state limiting the generalisability of the findings. |
| Mark, Rabiu & Santas, (2023) | The study evaluated the health communication strategies of the Ministry of Health in Benue, Plateau and Nasarawa state. | A survey was used for those parents who have had experience with child immunisation before; focus group discussion was used for nursing mothers; and caregivers; while in-depth interview was used for those health who have been on the immunisation beat.  Survey comprising 376 respondents, 3 Focus  Group Discussion comprising 6 participants each, and in-depth interview. | Mixed method design. | Key findings of the research from the survey were that the extent of awareness of the child immunisation campaign was due to the role played by influential religious/traditional leaders; and that ignorance and illiteracy were major challenges of the child immunisation campaign. Concerning the findings from the in-depth interview, the respondents (health workers) noted that their key communication strategies included community stakeholders, such as religious/traditional leaders, and complementing that with the mass media. | Involved a ranged of stakeholders. | Small sample size. |
| Oku et al., (2017) | The study explored experiences of caregivers and health workers in Nigeria on vaccination communication strategies implemented in their settings. | The study adopted observations (n = 40), in-depth interviews (n = 14) and focus group  discussions (FGDs) (n = 12) amongst 14 purposively selected health workers, two community leaders and 84 caregivers in Bauchi and Cross river states. | Qualitative methods. | The study found that caregivers were informed about vaccination activities through health facilities (during health education sessions conducted at antenatal or immunization clinics), media outlets; and announcements (in churches/mosques, communities and markets). . Some caregivers perceived the clinic environment, long waiting times and health worker attitudes as barriers to receiving vaccination information. | Involved a ranged of stakeholders. The study’s inclusion of two Nigerian states and both urban and rural sites in those states, allowed for the gaining of more complete picture of vaccination communication issues. | The study was not conducted in the northern states with the lowest immunization uptake, or where uptake rates may be lower. Also, there is likely a degree of  selection bias in the focus groups as caregivers who did not (or could not) attend the vaccination clinic did not have the opportunity to participate in the study |
| Oladepo, Dipeolu & Oladunni (2020) | The study examined the effect of SMS reminder sent to mothers in rural communities on full and timely completion of routine childhood immunization. | 3500 mothers of infants were categorized into the Intervention and Control groups recruited at various Primary Healthcare Centres in 6 states and the FCT, Nigeria. | Quasi-experimental design. | The study found a significant association between adherence to appointment dates and completeness of routine immunization vaccine was found. The Intervention group had a significantly higher completion rate for measles and yellow fever vaccines (55.3%; 75.9%) compared with the Control group (26.8%; 23.9%). | The study strength lies in enrolling mothers of infants at health facilities, selection bias was minimized compared with what would have been if they were enrolled in their various homes; bearing in mind that routine immunization can only be given at health facilities irrespective of where a woman delivered her baby. | The authors could not monitor the delivery status of the SMS messages as it was unidirectional. It will be desirable in future studies that a link of the delivery status of sent SMS messages should be established to monitor actual delivery to individual receiver. |
| Oyo-Ita et al., (2020) | The study aim was to measure the impact of engaging the traditional and religious leaders in influencing vaccination uptake in Cross River State, Nigeria. | A total of 2598, 2570, and 2550 children were assessed at baseline, midterm, and endline respectively. | Experimental design. | The results showed that the intervention had no evidence of impact on the proportion of children with up-to-date with vaccination (p=0.69). However, it was effective in reducing the number of unvaccinated children from 7% to 0.4% (p=0.001). It was also effective in improving timeliness of the later vaccines Penta 3 (OR 1.55; 95% CI: 1.14, 2.12; p= 0.005) and Measles (OR 2.81; 96% CI: 1.93-4.1; p | Large sample size. | The study focused on only one state in Nigeria. |
| Oyo-Ita et al., (2021) | This study assessed the cost-effectiveness of using a multi-faceted intervention  that included traditional and religious leaders for community engagement to improve uptake of routine immunisation services in communities in Cross River State, | Three wards were randomly selected from each LGA, and 4 villages from each ward making a total of 48 villages per study arm comprising traditional leaders. | Cluster-randomized controlled trial. | The cost-effectiveness analysis showed that the incremental cost of the initial implementation of the intervention was US$19,357and that the incremental effect was 323 measles cases averted, resulting in an incremental cost-effectiveness ratio (ICER) of US$60/measles case averted. The study found that traditional and religious leaders’ involvement in vaccination is a cost-effective strategy for improving the uptake of childhood routine vaccinations. | Large sample size. | The study focused on only one state in Nigeria. |
| Ozawa et al., (2018) | This study aimed to understand parents’ preferences and identify possible interventions to improve uptake of childhood immunization. | 97 men and 101 women in 198 households were surveyed in Zamfara state. | Quantitative study. | The study found that routine immunization programs are encouraged to consider joining forces with food and nutritional programs to improve vaccination uptake. Incorporating door-to-door visits and media campaigns to target older and unemployed populations may increase childhood immunization uptake | The study strength lies in its eliciting participants preferences to inform health policy | The study reported self-reported data, which may be subject to social desirability bias. |
| Promise et al., (2025) | This study evaluates the impact of mobile phone (MP) reminders and home visits (HV) on childhood immunization completion rates in Bayelsa State, Nigeria. | A total of 384 participants were randomly assigned to three study arms: control (no intervention), MP reminders, and HV outreach (128 per arm). | Pragmatic cluster randomized controlled trial. | The study revealed significant differences in immunization completion rates across study groups: control (46.1%, 59/128), MP reminders (59.4%, 76/128), and HV outreach (63.3%, 81/128). Both interventions were effective in improving vaccination uptake, with a statistically significant difference (p = 0.015) and a moderate effect size (0.257). However, HV outreach demonstrated the highest completion rate, suggesting its greater effectiveness in overcoming access and engagement barriers. | Large sample size drawn from Bayelsa state. | The study only focused on one state and might not form the basis for generalisation. |
| Udochi‑Nwachukwu, Dada, & Nyashanu, (2023). | The study examined the use of indigenous communication media for immunization promotion among mothers in rural communities of Abia State, Nigeria. | 394 community participants comprising males and females across 10 rural communities in Abia State. | Quantitative study incorporating surveys. | The findings found that 53.8%, 25.6%, and 20.6% of the participants averred that indigenous communication media played the role of immunization information dissemination, reminder, and reinforcement, respectively. Most (73.6%) of the respondents opined that indigenous communication media are highly relevant in informing rural dwellers about immunization programs. | Large sample size drawn from Abia state. | The study only focused on one state and might not form the basis for generalisation. |
| Yunusa et al., (2022) | The study examined the effect of mobile phone text message and call reminders in the completeness of the pentavalent vaccine administered against pertussis, diphtheria, hepatitis B, tetanus and Haemophilus influenza. | A total of 541 mothers of children not more than three weeks old  (271 in the intervention group and 270 in the control group) participated in the study and was recruited from Kano state, | Quasi-experimental study. | The study found that completion rates for the three doses of the pentavalent vaccine were observed to be higher for children in the reminder group (n = 161, 59.4%) compared to those in the control group (n = 92, 34.1%). | Large sample size. | The study assessed the effectiveness of mobile phone reminders in the completeness of only the pentavalent vaccine. It did not examine the effect of the mobile technology on other routine childhood vaccines like Oral polio vaccine and BCG. |

**Appendix 2: The Joanna Briggs Institute (JBI) Critical Appraisal Checklist for Qualitative Research**

| Author(s) | 1. Is there congruity between the stated philosophical perspective and the research methodology?  Yes/No/Unclear/ Not applicable | 2. Is there congruity between the research methodology and the research question or objectives? Yes/No/Unclear/ Not applicable | 3. Is there congruity between the research methodology and the methods used to collect data? Yes/No/Unclear/ Not applicable | 4. Is there congruity between the research methodology and the representation and analysis of data? Yes/No/Unclear/ Not applicable | 5. Is there congruity between the research methodology and the interpretation of results? Yes/No/Unclear/ Not applicable | 6. Is there a statement locating the researcher culturally or theoretically? Yes/No/Unclear/ Not applicable | 7. Is the influence of the researcher on the research, and vice- versa, addressed? Yes/No/Unclear/ Not applicable | 8. Are participants, and their voices, adequately represented? Yes/No/Unclear/ Not applicable | 9. Is the research ethical according to current criteria or, for recent studies, and is there evidence of ethical approval by an appropriate body? Yes/No/Unclear/ Not applicable | 10. Do the conclusions drawn in the research report flow from the analysis, or interpretation, of the data? Yes/No/Unclear/ Not applicable | 11. Overall appraisal: include, exclude, seek further info | 12.Comments including reasons for exclusion |
| --- | --- | --- | --- | --- | --- | --- | --- | --- | --- | --- | --- | --- |
| Oku et al., (2017) | Unclear | Yes | Yes | Yes | Yes | No | No | Yes | Yes | Yes | Include | The research focussed on the target population and met the review inclusion criteria. |

**Appendix 3: Quality Appraisal Checklist for Quantitative, Qualitative, and Mixed Methods Studies (Symonds & Tang et al., 2024)**

| Author(s) | 1. Was the study setting (venues) and/or geographic location described in detail? | 2. Were the recruitment processes explained? | 3. Was the administration process explained? | 4. Did the researchers receive informed consent from human participants? | 5. Is there a clear statement about ethical practices? | 6. Was the study sample described in detail? | 7. Were the numbers of people who did/did not consent to participate stated? | 8. Was the number of participants justified? | 9. Was the missingness of data mentioned? | 10. Was the number of participants at each stage/ wave specified? | 11. Did the authors report their missing data handling strategy? | 12. Was the reliability and validity of measurement methods mentioned? | 13.  For studies with repeated measures / longitudinal data |
| --- | --- | --- | --- | --- | --- | --- | --- | --- | --- | --- | --- | --- | --- |
| Kaka, Igvuve & Santas, (2026) | Yes | Yes | Yes | Yes | Yes | Yes | Yes | Yes | Unclear | Not applicable (N/A) | Not clear | Yes | N/A |
| Mark, Rabiu & Santas, (2023) | Yes | Yes | Yes | Yes | Yes | Yes | Yes | Yes | Unclear | Not applicable (N/A) | Not clear | Yes | N/A |
| Ozawa et al., (2018) | Yes | Yes | Yes | Yes | Yes | Yes | Yes | Yes | Unclear | Not applicable (N/A) | Not clear | Yes | N/A |
| Udochi‑Nwachukwu, Dada, & Nyashanu, (2023). | Yes | Yes | Yes | Yes | Yes | Yes | Yes | Yes | Unclear | Not applicable (N/A) | No | Yes |  |

**Appendix 4: CASP informed Quality and Risk of Bias Assessment of Included Studies (Mixed method)**

| Article title and year | 1. Was there a clear statement of the aims of the research? | 2. Is a qualitative/quantitative methodology appropriate? | 3. Was the research design appropriate to address the aims of the research? | 4. Was the recruitment strategy appropriate to the aims of the research? | 5. Was the data collected in a way that addressed the research issue? | 6. Has the relationship between the researcher and participants been adequately considered? | 7. Have ethical issues been taken into consideration? | 8. Was the data analysis sufficiently rigorous? | 9. Is there a clear statement of findings? | 10. How valuable is the research? |
| --- | --- | --- | --- | --- | --- | --- | --- | --- | --- | --- |
| Iwu et al., (2025) | Yes | Yes | Yes | Yes | Yes | Unclear | Yes | Yes | Yes | Very valuable. |

**Appendix 5: JBI Critical Appraisal Checklist for Randomized Controlled Trials**

| Article author and year | 1. Was true randomization used for assignment of participants to treatment groups? | 2. Was allocation to treatment groups concealed? | 3. Were treatment groups similar at the baseline? | 4. Were participants blind to treatment assignment? | 5. . Were those delivering treatment blind to treatment assignment? | 6. . Were outcomes assessors blind to treatment assignment? | 7. Were treatment groups treated identically other than the intervention of interest? | 8. Was follow up complete and if not, were differences between groups in terms of their follow up adequately described and analyzed? | 9. Were participants analyzed in the groups to which they were randomized? | 10. Were outcomes measured in the same way for treatment groups? | 11. Were outcomes measured in a reliable way? | 12. Was appropriate statistical analysis used? | 13. Was the trial design appropriate, and any deviations from the standard RCT design (individual randomization, parallel groups) accounted for in the conduct and analysis of the trial? | Overall appraisal: |
| --- | --- | --- | --- | --- | --- | --- | --- | --- | --- | --- | --- | --- | --- | --- |
| Brown & Oluwatosin, (2017) | Yes | Unclear | Yes | Unclear | Unclear | Unclear | Yes | Yes | Yes | Yes | Yes | Yes | Yes | Include |
| Oyo-Ita et al., (2020) | Yes | Yes | Yes | No | No | Yes | Yes | Yes | Yes | Yes | Yes | Yes | Yes | Include |
| Oyo-Ita et al., (2021) | Yes | Yes | Yes | Yes | No | Yes | Yes | Yes | Yes | Yes | Yes | Yes | Yes | Include |
| Oladepo, Dipeolu & Oladunni (2020) | Yes | Unclear | Yes | Unclear | Unclear | Unclear | Yes | N/A | Yes | Yes | Yes | Yes | Yes | Include |
| Promise et al., (2018) | Yes | Yes | Yes | Yes | Yes | Unclear | Yes | Yes | Yes | Yes | Yes | Yes | Yes | Include |
| Yunusa et al., (2022) | Yes | Unclear | Yes | Unclear | Unclear | Unclear | Yes | N/A | Yes | Yes | Yes | Yes | Yes | Include |

**Appendix 6: PRISMA Checklist**

| **Section and Topic** | **Item #** | **Checklist item** | **Location where item is reported** |
| --- | --- | --- | --- |
| **TITLE** | | |  |
| Title | 1 | Identify the report as a systematic review. | Title and abstract |
| **ABSTRACT** | | |  |
| Abstract | 2 | See the PRISMA 2020 for Abstracts checklist. | Abstract |
| **INTRODUCTION** | | |  |
| Rationale | 3 | Describe the rationale for the review in the context of existing knowledge. | Introduction |
| Objectives | 4 | Provide an explicit statement of the objective(s) or question(s) the review addresses. | Introduction |
| **METHODS** | | |  |
| Eligibility criteria | 5 | Specify the inclusion and exclusion criteria for the review and how studies were grouped for the syntheses. | Methods |
| Information sources | 6 | Specify all databases, registers, websites, organisations, reference lists and other sources searched or consulted to identify studies. Specify the date when each source was last searched or consulted. | Methods |
| Search strategy | 7 | Present the full search strategies for all databases, registers and websites, including any filters and limits used. | Methods |
| Selection process | 8 | Specify the methods used to decide whether a study met the inclusion criteria of the review, including how many reviewers screened each record and each report retrieved, whether they worked independently, and if applicable, details of automation tools used in the process. | Methods |
| Data collection process | 9 | Specify the methods used to collect data from reports, including how many reviewers collected data from each report, whether they worked independently, any processes for obtaining or confirming data from study investigators, and if applicable, details of automation tools used in the process. | Methods |
| Data items | 10a | List and define all outcomes for which data were sought. Specify whether all results that were compatible with each outcome domain in each study were sought (e.g. for all measures, time points, analyses), and if not, the methods used to decide which results to collect. | Methods |
|  | 10b | List and define all other variables for which data were sought (e.g. participant and intervention characteristics, funding sources). Describe any assumptions made about any missing or unclear information. | Methods and Appendix |
| Study risk of bias assessment | 11 | Specify the methods used to assess risk of bias in the included studies, including details of the tool(s) used, how many reviewers assessed each study and whether they worked independently, and if applicable, details of automation tools used in the process. | Methods and Appendix |
| Effect measures | 12 | Specify for each outcome the effect measure(s) (e.g. risk ratio, mean difference) used in the synthesis or presentation of results. | Not applicable |
| Synthesis methods | 13a | Describe the processes used to decide which studies were eligible for each synthesis (e.g. tabulating the study intervention characteristics and comparing against the planned groups for each synthesis (item #5)). | Methods and appendix |
|  | 13b | Describe any methods required to prepare the data for presentation or synthesis, such as handling of missing summary statistics, or data conversions. | Not applicable |
|  | 13c | Describe any methods used to tabulate or visually display results of individual studies and syntheses. | Methods and appendix |
|  | 13d | Describe any methods used to synthesize results and provide a rationale for the choice(s). If meta-analysis was performed, describe the model(s), method(s) to identify the presence and extent of statistical heterogeneity, and software package(s) used. | Methods and appendix |
|  | 13e | Describe any methods used to explore possible causes of heterogeneity among study results (e.g. subgroup analysis, meta-regression). | Not applicable |
|  | 13f | Describe any sensitivity analyses conducted to assess robustness of the synthesized results. | Methods and appendix |
| Reporting bias assessment | 14 | Describe any methods used to assess risk of bias due to missing results in a synthesis (arising from reporting biases). | Methods and appendix |
| Certainty assessment | 15 | Describe any methods used to assess certainty (or confidence) in the body of evidence for an outcome. | Methods and appendix |
| **RESULTS** | | |  |
| Study selection | 16a | Describe the results of the search and selection process, from the number of records identified in the search to the number of studies included in the review, ideally using a flow diagram. | Methods and appendix |
|  | 16b | Cite studies that might appear to meet the inclusion criteria, but which were excluded, and explain why they were excluded. | Methods |
| Study characteristics | 17 | Cite each included study and present its characteristics. | Methods, findings and appendix |
| Risk of bias in studies | 18 | Present assessments of risk of bias for each included study. | Methods and appendix |
| Results of individual studies | 19 | For all outcomes, present, for each study: (a) summary statistics for each group (where appropriate) and (b) an effect estimate and its precision (e.g. confidence/credible interval), ideally using structured tables or plots. | Not applicable |
| Results of syntheses | 20a | For each synthesis, briefly summarise the characteristics and risk of bias among contributing studies. | Methods and appendix |
|  | 20b | Present results of all statistical syntheses conducted. If meta-analysis was done, present for each the summary estimate and its precision (e.g. confidence/credible interval) and measures of statistical heterogeneity. If comparing groups, describe the direction of the effect. | Findings |
|  | 20c | Present results of all investigations of possible causes of heterogeneity among study results. | Not applicable |
|  | 20d | Present results of all sensitivity analyses conducted to assess the robustness of the synthesized results. | Methods and appendix |
| Reporting biases | 21 | Present assessments of risk of bias due to missing results (arising from reporting biases) for each synthesis assessed. | Methods and appendix |
| Certainty of evidence | 22 | Present assessments of certainty (or confidence) in the body of evidence for each outcome assessed. | Methods and appendix |
| **DISCUSSION** | | |  |
| Discussion | 23a | Provide a general interpretation of the results in the context of other evidence. | Findings and discussion |
|  | 23b | Discuss any limitations of the evidence included in the review. | Discussion |
|  | 23c | Discuss any limitations of the review processes used. | Discussion |
|  | 23d | Discuss implications of the results for practice, policy, and future research. | Discussion |
| **OTHER INFORMATION** | | |  |
| Registration and protocol | 24a | Provide registration information for the review, including register name and registration number, or state that the review was not registered. | Abstract and methods |
|  | 24b | Indicate where the review protocol can be accessed, or state that a protocol was not prepared. | Abstract and methods |
|  | 24c | Describe and explain any amendments to information provided at registration or in the protocol. | Not applicable |
| Support | 25 | Describe sources of financial or non-financial support for the review, and the role of the funders or sponsors in the review. | Not applicable |
| Competing interests | 26 | Declare any competing interests of review authors. | Not applicable |
| Availability of data, code and other materials | 27 | Report which of the following are publicly available and where they can be found: template data collection forms; data extracted from included studies; data used for all analyses; analytic code; any other materials used in the review. | Methods and discussion |

**Appendix 7 – Definition of terms**

| Terms | Definition |
| --- | --- |
| State sponsored communication strategies | State-sponsored communication strategies are deliberate, government-led campaigns designed to inform the public, shape domestic behaviour, and influence behaviour change. such campaign is also designed to promote public understanding and drive behavioural change (e.g., public health campaigns). |
| Community-centered interventions | Community-centred interventions refer to programs and initiatives that aim to improve the health and well-being of specific population groups within a defined local community. These interventions are often multicomponent, meaning that they employ multiple strategies and tactics to achieve their goals. (Hallie E. Ford Center, 2026) |
| Phone based interventions | Telephone interventions are healthcare strategies delivered via phone calls, aimed at providing education, support, or specific health-related services to individuals, often used to assist caregivers or manage health conditions remotely (Andrades-González & Molina-Mula, 2022). |
| Media campaign interventions | Media campaign interventions are defined as interventions that generally consist of brief, recurring messages, disseminated through broadcast, Internet, and print media, to inform and motivate individuals and foster behaviour change whilst creating awareness (Faggiano & Vigna-Taglianti, 2008). |
| Health belief model | The **Health Belief Model (HBM)** is defined as psychological framework that explains health-related behavior based on factors such as perceived susceptibility, perceived severity, perceived benefits, perceived barriers, cues to action, and self-efficacy (Rosenstock, 1974). |
